# Implementation of a novel population panel management curriculum among interprofessional health care trainees

**DOI:** 10.1186/s12909-017-1093-y

**Published:** 2017-12-22

**Authors:** Catherine P. Kaminetzky, Lauren A. Beste, Anne P. Poppe, Daniel B. Doan, Howard K. Mun, Nancy Fugate Woods, Joyce E. Wipf

**Affiliations:** 1 VA Puget Sound Health Care System, Seattle, WA USA; 2 Division of General Internal Medicine, University of Washington, Seattle, WA USA; 3 Biobehavioral Nursing and Health Informatics, University of Washington School of Nursing, Seattle, WA USA; 4 Providence Health System, Seattle, WA USA; 5 de Tornyay Center for Healthy Aging, University of Washington School of Nursing, Seattle, WA USA

**Keywords:** Panel management, Graduate medical education, Interprofessional education, Chronic disease management, Population health

## Abstract

**Background:**

Gaps in chronic disease management have led to calls for novel methods of interprofessional, team-based care. Population panel management (PPM), the process of continuous quality improvement across groups of patients, is rarely included in health professions training for physicians, nurses, or pharmacists. The feasibility and acceptance of such training across different healthcare professions is unknown. We developed and implemented a novel, interprofessional PPM curriculum targeted to diverse health professions trainees.

**Methods:**

The curriculum was implemented annually among internal medicine residents, nurse practitioner students and residents, and pharmacy residents co-located in a large, academic primary care site. Small groups of interprofessional trainees participated in supervised quarterly seminars focusing on chronic disease management (e.g., diabetes mellitus, hypertension, or chronic obstructive pulmonary disease) or processes of care (e.g., emergency department utilization for nonacute conditions or chronic opioid management). Following brief didactic presentations, trainees self-assessed their clinic performance using patient-level chart review, presented individual cases to interprofessional staff and faculty, and implemented subsequent feedback with their clinic team. We report data from 2011 to 2015. Program evaluation included post-session participant surveys regarding attitudes, knowledge and confidence towards PPM, ability to identify patients for referral to interprofessional team members, and major learning points from the session. Directed content analysis was performed on an open-ended survey question.

**Results:**

Trainees (*n* = 168) completed 122 evaluation assessments. Trainees overwhelmingly reported increased confidence in using PPM and increased knowledge about managing their patient panel. Trainees reported improved ability to identify patients who would benefit from multidisciplinary care or referral to another team member. Directed content analysis revealed that trainees viewed team members as important system resources (*n* = 82).

**Conclusions:**

Structured interprofessional training in PPM is both feasible and acceptable to trainees across multiple professions. Curriculum participants reported improved panel management skills, increased confidence in using PPM, and increased confidence in identifying candidates for interprofessional care. The curriculum could be readily exported to other programs and contexts.

**Electronic supplementary material:**

The online version of this article (10.1186/s12909-017-1093-y) contains supplementary material, which is available to authorized users.

## Background

The National Academy of Medicine endorses interprofessional chronic disease management as paramount to addressing quality gaps, yet formal training in team-based approaches rarely occurs during healthcare professional education [1]. In addition, significant care gaps exist in management of chronic diseases, such as diabetes mellitus, [2] hypertension, [3] and chronic obstructive disease [4]. Experiential curricula are needed to prepare future primary care providers to adopt a population-focused practice, [5] deliver high-quality chronic disease management, [5–7] and function on interprofessional health care teams [8–10].

Population panel management (PPM), a form of population health management, is “a set of tools and processes for population care that are applied systematically at the level of the primary care panel” [11]. PPM utilizes data to identify patients with unmet preventive and chronic disease care needs, [11, 12] allowing quality care to be provided for all empaneled patients, regardless of how frequently they present for in-person care. Effective PPM requires an understanding of system resources and how to apply them to individual patients, both during and between visits. PPM has been associated with improved care quality for a variety of health conditions, [13–16] and is associated with lower burnout and cynicism in primary care providers [17]. Primary care staff report increased panel management efficacy with training [18]. Trainees participating in interprofessional, case-based PPM conferences have reported improvements in developing treatment plans, [19] but little is known about whether PPM enhances interprofessional practice nor the feasibility and acceptance of such training across professions.

Using evidence-based curriculum development principles, [20] an interprofessional group of educators developed a novel PPM curriculum targeted to physician, nurse, and pharmacy trainees. This paper describes the feasibility and acceptability of the workplace-based interprofessional PPM curriculum. The objectives of this analysis were to assess the following trainee-reported outcomes as a result of the curriculum: 1) confidence in utilizing PPM tools to assess clinical performance, 2) ability to work with interprofessional team members to overcome gaps in care, and 3) understanding of system resources available for chronic disease management.

## Methods

### Setting and participants

In 2011, the Veterans Affairs (VA) Office of Academic Affiliations created VA Centers of Excellence in Primary Care Education (CoEPCE) with a mission to transform primary care training within VA’s patient centered medical home practices [21]. Our CoEPCE primary care teams typically consist of a trainee provider (physician or nurse practitioner), supervising provider, nurse care manager, licensed practical nurse or health care technician, clinical pharmacist, pharmacy resident, and clerical assistant, and are thus ideally suited for interprofessional collaboration. Our CoEPCE is a cooperative effort between a large, tertiary VA facility and Schools of Nursing and Medicine at a large, academic institution. Our CoEPCE hosts internal medicine residents, doctorate of nursing practice (DNP) students, nurse practitioner residents, pharmacy residents and other health professions trainees in our academic Primary Care and Women’s Clinics serving 1250 patients on CoEPCE teams. Physician residents request to participate in CoEPCE after they match to internal medicine residency, prior to the start of their intern year. Nurse practitioner students have the opportunity to apply to the CoEPCE after admission to their nursing program; and nurse practitioner residents apply directly to the CoEPCE.

### Program description

#### Context and needs assessment

Appraisal of existing curricula within our respective programs (internal medicine residency, nursing practice, and pharmacy) demonstrated that each offered quality improvement content, but none utilized population-based data reviews to analyze trainee clinical performance. Discussions with trainee and faculty stakeholders revealed that, while our facility provided disease registries for selected conditions (e.g., diabetes), few trainees or providers knew of their existence or how to utilize them to effect practice change. In addition, trainees had varying knowledge of system resources that could be applied to improve chronic disease management. Formal surveys of Internal Medicine Residency Program graduates indicated a desire for greater confidence in outpatient management of chronic diseases. Finally, facility leadership indicated strong support for improving the use of data to improve patient care and enhanced interprofessional collaboration within primary care teams.

#### Goals

The overall goal of the PPM curriculum is to improve patient care within an interprofessional practice using a systematic, data-driven approach to identify areas for improvement in primary care focused conditions and processes of care. Program objectives of the PPM curriculum are to: 1) utilize disease registries and other sources of performance data to identify gaps in preventive health and chronic disease care; 2) develop and implement patient-focused action plans; and 3) effectively work with health care team members to improve individual patient care.

#### Curriculum development and piloting

We developed a year-long PPM curriculum focusing on clinical knowledge, system resources, and interprofessional collaboration. Early pilot sessions revealed a general lack of understanding of the roles and scopes of practice of interprofessional care among team members in our clinic. Attempts to address this through lecture proved unpalatable to trainees. Drawing on Kolb’s experiential learning model and situated learning principles, which hold that learners are socialized where they learn, [22] the final PPM curriculum utilized small group discussion format, with trainees performing a chart review of their own patients and formally presenting cases to interprofessional trainees and faculty (clinical pharmacist, registered nurse care manager, physician, and staff and faculty from other professions).

PPM sessions were conducted quarterly. Each 2–3 h session included 3–12 trainees and was co-facilitated by an attending physician and a clinical pharmacy specialist. Each session focused on a chronic condition (e.g., diabetes mellitus, hypertension, or chronic obstructive pulmonary disease) or process of care (e.g., emergency department utilization for nonacute conditions or chronic opioid management). Sessions followed a standard flow (Table 1). Following 15 to 30 min of didactic content, participants brainstormed relevant indicators of quality care, reached consensus on the quality indicators to include in the chart review, and created a tool to facilitate targeted chart review (Table 2). Trainees received a list of applicable patients from their own panel and performed 2–4 detailed chart reviews using the electronic health record (EHR).

**Table 1:** Population panel management session flow

Step	Time allotted	Content covered
1	15–30 min	Didactic addressing chronic disease or process of care
2	15–20 min	Brainstorm indicators for quality, reach consensus on items to include in chart review and create chart review tool
3	30–40 min	Participants review the clinical data of 2–4 of their own patients with relevant care gaps
4	60 min	Participants take turns presenting cases to interprofessional team and take action in the EHR based on audience feedback: writing orders, notifying team members of needed actions and sending letters to patients.
5	5 min	Participants write three observations for improvement and complete post-session evaluation

**Table 2:** Sample chart review tool from session on diabetes mellitus type 2

Chronic disease data	Patient A data	Patient B data
Patient Identifier		
Date of Last visit		
Age		
Comorbidities (Coronary Artery Disease, Stroke, Hypertension)		
Tobacco		
Hemoglobin A1c / date		
Medications		
Medications refilled as expected?		
Last blood pressure / date		
Body Mass Index		
Urine microalbumin / date		
LDL cholesterol / date		
Dilated retinal exam result / date		

Write three observations for improvement: 1 2 3

Learners presented cases to each other and to the interprofessional faculty team. The group contributed management strategies and suggestions for additional resources, and presenters took appropriate action in real-time utilizing the EHR (e.g., ordering consults or medications, sending letters to patients, notifying team members of needed tasks). To connect PPM to future quality improvement projects, sessions concluded with group reflection on areas for improvement in individual and system practice.

#### Program feasibility

Faculty time for curriculum development was supported by funding from VA’s Office of Academic Affiliations. Trainee time was incorporated as part of ambulatory clinic rotations. Additional staff or trainees from social work, psychology, psychiatry, and nursing participated as co-facilitators on an ad hoc basis. PPM sessions were held in a computer lab to facilitate access to data resources and the EHR.

Trainees received a list of candidates for chart review before each PPM session. Initially, these were compiled by a data manager. However, as disease- and process-specific registries became available at our facility, trainees accessed their own performance data in real time.

### Program evaluation

We developed a 13-item post-session evaluation instrument [Additional file 1] to assess three constructs: perception of the curricular content, confidence in performing PPM, and likelihood of using the techniques in the future. Seven items assessed trainee perceptions using a six-point Likert scale with responses ranging from 0 (not at all) to 5 (very much). Trainees utilized a visual analog scale to rate their increase in confidence in using PPM (Range: 0 (not at all) to 5 (very much)). To assess fidelity of the curricular sessions to the course objectives, an open-ended question asked “What are the major take-home lessons for you today?” To seek input for continuous improvement, an open question asked “What would you suggest for improvement?” The survey was administered anonymously at the end of each session. Mean scores and standard deviation were calculated. Data from incomplete surveys were included for each complete response. All analyses were conducted using MS Office Excel 2010.

Three authors (CK, AP, JW) performed directed content analysis on open-ended responses to the question regarding take-home lessons from the session. Directed content analysis allows for validation and extension of a theoretical framework: data is analyzed using predetermined codes, while new codes are developed to define text not fitting the initial coding scheme. [23] We used the following initial coding categories: PPM, system resources, and clinical knowledge. Text data were reviewed and coded independently. Data that could not be coded using predefined themes were reviewed collaboratively to identify new categories and subcategories. We developed a code book to define themes and log exemplars. Discrepancies between reviewers were adjudicated until consensus was reached. Our evaluation was exempted by the institutional review board of the University of Washington and considered educational operations by the institutional review board of the VA facility.

## Results

Between 2011 and 2015, 168 trainees participated in 45 PPM sessions*.* One hundred twenty-two evaluation surveys were completed by 84 physician residents, 30 nurse practitioner students, and 8 pharmacy residents (Table 3). Eight surveys contained incomplete data.

**Table 3:** Learners’ Mean (SD) rating of population panel management sessions*

	Physician residents	Nurse practitioner students	Pharmacy residents	Total
	(*n* = 84)	(*n* = 30)	(*n* = 8)	(*n* = 122)
Was the content useful to your practice?	4.3 (0.68)	4.8 (0.41)	4.1 (0.99)	4.4 (0.69)
Did the content increase your knowledge about managing your patient panel?	4.2 (0.74)	4.7 (0.55)	4.4 (0.74)	4.3 (0.73)
How likely are you to use the content?	4.3 (0.74)	4.7 (0.52)	4.3 (0.89)	4.4 (0.72)
I can identify patients from my panel who would benefit from working with another care team member.	4.2 (0.86)	4.3 (0.66)	4.0 (0.58)	4.2 (0.80)
I can identify patients from my panel who would benefit from coordinated care by the care team.	4.3 (0.79)	4.4 (0.63)	4.0 (0.58)	4.3 (0.74)
I feel like I have accomplished something worthwhile.	4.2 (0.79)	4.5 (0.73)	4.1 (0.64)	4.2 (0.77)
I feel I’m positively influencing care of patients on my panel.	4.1 (0.80)	4.4 (0.63)	4.1 (0.64)	4.2 (0.76)

*The learners used a six-point scale where 0 = "Not at all" and 5 = "Very Much"

Overall, learners rated PPM’s usefulness to their practice with a mean of 4.4 (SD 0.69) on a scale ranging from 0 to 5. Learners reported PPM increased their knowledge about managing their panel with a mean of 4.3 (SD 0.73). They reported being able to identify patients who would benefit from working with another care team member, and being able to identify patients who would benefit from coordinated care with a mean of 4.2 (SD 0.80) and 4.3 (SD 0.74), respectively. Trainees rated the likelihood of using the content with a mean of 4.4 (SD 0.72); accomplishing something worthwhile with a mean of 4.2 (SD 0.77); and they rated positively influencing the care of their patients with a mean of 4.2 (SD 0.76). Learners reported increased confidence in using tools to enhance patient care, with a mean of 3.7 (SD 0.82).

Eight-two surveys (49% of respondents) included responses to the free response question “*What are the major take-home lessons for you today?”* Directed content analysis confirmed pre-identified categories of interest (Table 4). Population panel management was mentioned in 56% of responses, such as “Good to routinely go through a list of patients and find those that have fallen through the cracks. This kind of [P]PM time should be available to all providers.” System resources were mentioned in 72% of comments, such as “Use the WA PMP [Washington Prescription Monitoring Program]” and “Small changes; always try to address [obesity]; always mention MOVE [group-visit-based exercise and weight loss program]! Refer to nutritionists if appropriate.” Clinical knowledge was mentioned in 27% of responses, such as “Chlorthalidone good first line antihypertensive. Close follow-up when starting treatment.” Three new subcategories of system resources were identified. Data Tools, defined as disease registries and electronic health record, were mentioned in 37% of responses, such as “The Primary Care almanac [disease registry] is a useful tool.” Consultative services, defined as providers or clinical services not included in the CoEPCE primary care team, were mentioned in 17% of responses, such as “Take advantage of Pain Service.” Lastly, Immediate Team Members, defined as members of the CoEPCE primary care team, were mentioned 15% of responses, such as “Use pharmacists & nurses for care coordination instead of just PCP.” Trainees most commonly identified system resources as important to the curriculum.

**Table 4:** Themes identified from take-home lessons question. “ *What are the major take-home lessons for you today?”* ( *N* = 82)

Themes	Exemplar quotes	*N* (% of responses)
Population Panel Management		
(e.g., application, process or content)	“*Good to routinely go through a list of patients and find those that have fallen through the cracks. This kind of PM time should be available to all providers.”* *“It’s easier than I thought to follow up on diabetics.”*	46 (56%)
Systems Resources		
- Data Tools (e.g., disease registries, electronic health record, opioid prescription registry)	*“The Primary Care almanac is a useful tool.”* *“Use the WA PMP [Prescription Monitoring Program].”*	30 (37%)
- Immediate Team Members (e.g., nurse, clinical pharmacy specialist)	*“Use pharmacists & nurses for care coordination instead of just the PCP.”* *“Reasons pts go to ER are myriad. My team & I can prevent some of them.”*	12 (15%)
- Consultative Services (e.g., nutrition, Endocrinology Consult)	*“Small changes; always try to address [obesity]; always mention MOVE [group-visit-based exercise program]! Refer to nutritionists if appropriate.”* *“Take advantage of Pain Service.”*	14 (17%)
Clinical Knowledge		
(e.g., diagnosis, treatment, and/or management of disease or process of care)	*“Avoid mixing opioids.”* *“Chlorthalidone good first line antihypertensive. Close follow-up when starting treatment.”*	22 (27%)

Trainees provided suggestions for curriculum improvement throughout the implementation. For example, nurse practitioner students, who were initially assigned to work with specific faculty but were not assigned a specific panel of patients, requested their own patient panel, as is the case for medicine residents and nurse practitioner residents. Assigned patient panels were created for students, utilizing a database in a secure drive. Early iterations of PPM occurred monthly and repeated the topics of hypertension and diabetes mellitus quarterly; however, trainees recommended repeating topics less frequently and adding other topics. Trainees strongly recommended that every participant be allowed time to present his or her own cases, which was accomplished through diligent facilitation and through limiting the number of participants per session. Trainees also requested regular access to their PPM data outside of sessions to allow for independent review with other professions in the clinic setting.

## Discussion

Health professions trainees participating in a novel PPM curriculum report that it increased their confidence in using tools to improve patient care, increased their knowledge about managing their panel, and enhanced their ability to identify patients who would benefit from interprofessional care. Acceptance of the interprofessional, experiential context was high across all participating professions, including physician, nurse practitioner, and pharmacy trainees.

Leading an interprofessional health care team has been suggested as an entrustable professional activity integral to primary care practice [24]. Our PPM curriculum fosters interprofessionalism in two key ways: 1) interprofessional discussion of management strategies for challenging cases, and 2) deliberate curricular emphasis on exploration of system resources, including members of the interprofessional primary care team. Unlike other studies of PPM, which utilized medical assistants or nurses to perform limited PPM tasks, [25–27] our PPM activities were performed by primary care trainees. We intentionally chose this design based on prior reports that primary care providers may be reluctant to delegate work due to lack of confidence in other team members [28] and because primary care providers may lack understanding of their role in an interprofessional team [29, 30]. In PPM, case discussions in an interprofessional team setting was designed to provide opportunities for engagement with relevant health professions, to enhance understanding of other professions’ expertise and roles on the team, and to offer practical application of task delegation. The PPM process brings learners out of their profession-specific silos and into collaborative practice and integrated care.

The high observed level of participant acceptance likely results, in part, from use of sound educational theory in developing the curriculum. We employed a systematic approach described by Thomas et al. that includes: problem identification; needs assessment; developing goals and objectives; deploying educational strategies that were targeted to our interprofessional learners and goals; implementation; and evaluation and feedback [20]. As a result, PPM was implemented into already-existing ambulatory care experiences of trainees, which promoted acceptance by program directors and by clinical leaders. Our curriculum provided trainees with data about their own patients, allowed them to assess their own clinical practice and provided strategies to improve the care they provided.

An additional strength of the PPM curriculum was the use of chart review as a strategy to narrow gaps in care for primary care patients. Prior studies have shown benefits of chart review for trainees, including increasing a sense of “ownership” over patients, [31] increasing awareness of the utility of data for quality improvement, [31] and improving the quality of care [32, 33]. However, best methods to communicate feedback to trainees are unclear [34].

Our findings are subject to a number of potential limitations, including applicability to settings with limited panel data available. Disease registry information and electronic healthcare data on a panel of patients may not be available in all primary care settings or training programs. While use of EHRs is associated with better clinical processes and outcomes, as recently as 2012 fewer than half of physicians reported having a computerized system for patient population management [35]. However, we suspect these prior data do not reflect the recent movement of health care systems toward implementing EHRs and chronic disease registries, prompted in part by US federal healthcare reforms linking reimbursement to enhanced chronic disease management.

Other limitations should be considered. CoEPCE trainees may be more interested in primary care and in population health management than other health professions trainees and may be unusually receptive to a PPM curriculum. In addition, our curriculum was implemented at a single site and evaluated using a non-validated instrument. The composition of trainee professional groups across medicine, nursing, and pharmacy may vary in other locations. To be more fully generalizable, the curriculum should be replicated in a larger sample and in a more general group of trainees. In order to protect the confidentiality of respondents we were unable to collect complete trainee demographic characteristics. Some physician trainees participated in PPM sessions across several years of training, resulting in variable duration of participation among trainees. Lastly, the impact of PPM on patient outcomes was beyond the scope of this study and should be examined in future studies. Despite these limitations, we believe that the overwhelmingly positive response to the program is strong encouragement that interprofessional PPM training is both feasible to implement and acceptable to trainees.

## Conclusions

Health professions trainees participating in an interprofessional PPM curriculum reported increased confidence and skills in conducting panel management and increased confidence in identifying patients who would benefit from interprofessional care. Further study is needed to determine whether PPM improves patient outcomes and can be disseminated on a larger scale, and to examine whether positive trainee attitudes and understanding surrounding interprofessionalism persist longitudinally.

## Additional file

Additional file 1: COE-PCE panel management post-session evaluation form. (DOCX 34 kb)

## Abbreviations

CoEPCE: Centers of Excellence in Primary Care Education
EHR: Electronic Health Record
ER: Emergency Room
MD: Physician
NP: Nurse Practitioner
PCP: Primary Care Provider
PPM: Population panel management
SD: Standard Deviation
US: United States
VA: Veterans Affairs
WA PMP: Washington Prescription Monitoring Program

## References

[1] Kohn LT, Corrigan JM, Donaldson MS (1999). To err is human: building a safer health system.

[2] National Committee for Quality Assurance: Comprehensive diabetes care. http://www.ncqa.org/report-cards/health-plans/state-of-health-care-quality/2016-table-of-contents/diabetes-care (2016). Accessed 01 Oct 2017.

[3] Centers for Disease Control and Prevention (2011). Vital signs: prevalence, treatment, and control of hypertension--United States, 1999-2002 and 2005-2008. MMWR Morb Mortal Wkly Rep.

[4] Anzueto A (2010). Primary care management of chronic obstructive pulmonary disease to reduce exacerbations and their consequences. Am J Med Sci.

[5] Cusack CM, Knudson AD, Kronstadt JL, Singer RF, Brown AL. Practice-based population health: information technology to support transformation to proactive primary care. Rockville, MD: Agency for Healthcare Research and Quality. 2010.

[6] Accreditation Council for Graduate Medical Education: ACGME program requirements for graduate medical education in internal medicine. http://www.acgme.org/Portals/0/PFAssets/ProgramRequirements/140_internal_medicine_2017-07-01.pdf?ver=2017-06-30-083345-723 (2017). Accessed 01 Oct 2017.

[7] The National Organization of Nurse Practitioner Faculties: Nurse practitioner core competencies. https://c.ymcdn.com/sites/nonpf.site-ym.com/resource/resmgr/competencies/20170516_NPCoreCompsContentF.pdf (2017). Accessed 01 Oct 2017.

[8] Accreditation Council for Graduate Medical Education: ACGME common program requirements. http://www.acgme.org/Portals/0/PFAssets/ProgramRequirements/CPRs_2017-07-01.pdf (2017). Accessed 01 Oct 2017.

[9] Rieselbach RE, Feldstein DA, Lee PT, Nasca TJ, Rockey PH, Steinmann AF, Stone VE (2014). Ambulatory training for primary care general internists: innovation with the affordable care act in mind. J Graduate Med Educ.

[10] World Health Organization. Transforming and scaling up health professionals' education and training: World Health Organization guidelines (2013). http://apps.who.int/iris/bitstream/10665/93635/1/9789241506502_eng.pdf. Accessed 01 Oct 2017. 26042324

[11] Neuwirth EB, Schmittdiel JA, Tallman K, Bellows J. Understanding panel management: a comparative study of an emerging approach to population care. Permanente J. 2007;11:12–20. 10.7812/tpp/07-040 PMC3057714 21461107

[12] Kaminetzky CP, Nelson KM (2015). In the office and in-between: the role of panel management in primary care. J Gen Intern Med.

[13] Loo TS, Davis RB, Lipsitz LA, Irish J, Bates CK, Agarwal K, Markson L, Hamel MB (2011). Electronic medical record reminders and panel management to improve primary care of elderly patients. Arch Intern Med.

[14] Kanter M, Martinez O, Lindsay G, Andrews K, Denver C (2010). Proactive office encounter: a systematic approach to preventive and chronic care at every patient encounter. Permanente J.

[15] Kravetz JD, Walsh RF (2016). Team-based hypertension management to improve blood pressure control. J Primary Care Comm Health.

[16] Xiang AH, Martinez MP, Wang X, Joshua AP, Chung J, Thai M, Lindsay G, Kanter M, Jacobsen SJ (2015). A proactive diabetes panel management approach: can it work and how does it work in a health care delivery system?. Journal of Patient-Centered Research and Reviews.

[17] Willard-Grace R, Dubé K, Hessler D, O’Brien B, Earnest G, Gupta R, Shunk R, Grumbach K. Panel management, team culture, and worklife experience. Families, Systems, Health. 2015;33:231. 10.1037/fsh0000113 25730504

[18] Strauss SM, Jensen AE, Bennett K, Skursky N, Sherman SE, Schwartz MD (2015). Clinicians' panel management self-efficacy to support their patients' smoking cessation and hypertension control needs. Transl Behav Med.

[19] Weppner WG, Davis K, Sordahl J, Willis J, Fisher A, Brotman A, Tivis R, Gordon T, Smith CS (2016). Interprofessional care conferences for high-risk primary care patients. Acad Med.

[20] Thomas PA, Kern DE, Hughes MT, Chen BY. Curriculum development for medical education: a six-step approach. 3rd ed. Baltimore: Johns Hopkins University Press; 2016.

[21] Gilman SC, Chokshi DA, Bowen JL, Rugen KW, Cox M. Connecting the dots: Interprofessional health education and delivery system redesign at the Veterans Health Administration. Acad Med. 2014;89:1113–6. 10.1097/ACM.0000000000000312 24853198

[22] Mann KV (2011). Theoretical perspectives in medical education: past experience and future possibilities. Med Educ.

[23] Hsieh H-F, Shannon SE (2005). Three approaches to qualitative content analysis. Qual Health Res.

[24] Chang A, Bowen JL, Buranosky RA, Frankel RM, Ghosh N, Rosenblum MJ, Thompson S, Green ML (2013). Transforming primary care training—patient-centered medical home entrustable professional activities for internal medicine residents. J Gen Intern Med.

[25] Bodenheimer T (2011). Lessons from the trenches—a high-functioning primary care clinic. N Engl J Med.

[26] Schwartz MD, Jensen A, Wang B, Bennett K, Dembitzer A, Strauss S, Schoenthaler A, Gillespie C, Sherman S (2015). Panel management to improve smoking and hypertension outcomes by VA primary care teams: a cluster-randomized controlled trial. J Gen Intern Med.

[27] Watts SA, Lucatorto M (2014). A review of recent literature - nurse case managers in diabetes care: equivalent or better outcomes compared to primary care providers. Curr Diab Rep.

[28] Solimeo SL, Ono SS, Lampman MA, Paez MB, Stewart GL. The empowerment paradox as a central challenge to patient centered medical home implementation in the Veteran's Health Administration. J Interprofessional Care. 2014;29:26–33. 10.3109/13561820.2014.937480 25052920

[29] Reid RJ, Coleman K, Johnson EA, Fishman PA, Hsu C, Soman MP, Trescott CE, Erikson M, Larson EB. The Group Health medical home at year two: cost savings, higher patient satisfaction, and less burnout for providers. Health Aff. 2010;29:835–43. 10.1377/hlthaff.2010.0158 20439869

[30] Ladebue AC, Helfrich CD, Gerdes ZT, Fihn SD, Nelson KM, Sayre GG (2016). The experience of patient aligned care team (PACT) members. Health Care Manag Rev.

[31] Bernabeo EC, Conforti LN, Holmboe ES (2009). The impact of a preventive cardiology quality improvement intervention on residents and clinics: a qualitative exploration. Am J Med Qual.

[32] Asao K, Mansi IA, Banks D (2009). Improving quality in an internal medicine residency program through a peer medical record audit. Acad Med.

[33] Houston TK, Wall T, Allison JJ, Palonen K, Willett LL, Keife CI, Massie FS, Benton EC, Heudebert GR (2006). Implementing achievable benchmarks in preventive health: a controlled trial in residency education. Acad Med.

[34] Ivers N, Jamtvedt G, Flottorp S, Young JM, Odgaard-Jensen J, French SD, O’Brien MA, Johansen M, Grimshaw J, Oxman AD. Audit and feedback: effects on professional practice and healthcare outcomes. Cochrane Database of Systematic Rev. 2012;6. 10.1002/14651858.CD000259.pub3 PMC11338587 22696318

[35] DesRoches CM, Audet A-M, Painter M, Donelan K (2013). Meeting meaningful use criteria and managing patient populations: a national survey of practicing physicians. Ann Intern Med.

