# Supplementary material for: Implementation of a novel population panel management curriculum among interprofessional health care trainees
Source: BMC Med Educ. 2017 Dec 22;17:264. doi: 10.1186/s12909-017-1093-y (PMC5741920; doi:10.1186/s12909-017-1093-y)
Supplement: Additional file 1: — COE-PCE panel management post-session evaluation form. (DOCX 34 kb) [file 12909_2017_1093_MOESM1_ESM.docx]

**Supplement 1.** COE-PCE Panel Management Session Evaluation Form

| **Panel Management Topic:** |  |  | **Date of session:** |  |
| --- | --- | --- | --- | --- |

| **Your Discipline:** | ⃝ NP | | ⃝ MD | | ⃝ Pharmacy | | ⃝ Other: | |  |
| --- | --- | --- | --- | --- | --- | --- | --- | --- | --- |
| **Your Affiliation:** | | ⃝ CoE | | ⃝ Non-CoE | | ⃝ Other: | |  | |

| **Presenters:** | ⃝ MD | | ⃝ NP | | ⃝ Pharmacist | | ⃝ Other(s): | |  | |  |
| --- | --- | --- | --- | --- | --- | --- | --- | --- | --- | --- | --- |
| **Names of Presenters:** | |  | |  | |  | |  | |  | |

| **Please rate the content of today’s session:** | Not at All |  |  | Very Much |
| --- | --- | --- | --- | --- |
| Useful to your practice? | 0 1 2 3 4 5 | | | |
| Increase your knowledge about managing your patient panel? | 0 1 2 3 4 5 | | | |
| How likely are you to use the content? | 0 1 2 3 4 5 | | | |

| **After today’s session:** | Not at All |  |  | Very Much |
| --- | --- | --- | --- | --- |
| I can identify patients from my panel who would benefit from working with another care team member. | 0 1 2 3 4 5 | | | |
| I can identify patients from my panel who would benefit from coordinated care by the care team. | 0 1 2 3 4 5 | | | |
| I feel like I have accomplished something worthwhile. | 0 1 2 3 4 5 | | | |
| I feel I’m positively influencing care of patients on my panel. | 0 1 2 3 4 5 | | | |

| Please rate how much your confidence increased regarding using the tools or skills from the session today with a slash mark on the line: | | | | | | | |
| --- | --- | --- | --- | --- | --- | --- | --- |
| 0  \|  Not at all |  |  |  |  | 5  \|  Very Much |  |  |

What are the major take-home lessons for you today?

What questions remain?

Please provide specific comments about this session:

What would you suggest for improvement?

Other comments and recommendations?
